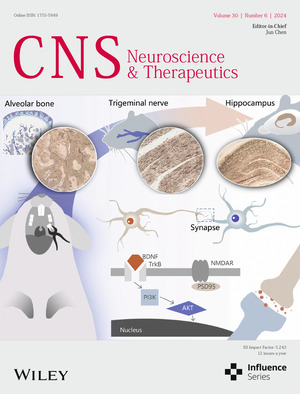# Additional Cover

**DOI:** 10.1111/cns.14837

**Published:** 2024-07-02

## Abstract

The cover image is based on the Original Article *Cognitive decline in Sprague–Dawley rats induced by neuroplasticity changes after occlusal support loss* by Xiaoyu Wang et al., https://doi.org/10.1111/cns.14750.